# Dual and Poly Use of Tobacco Products in a Sample of Pregnant Smokers: A Cross-sectional Study

**DOI:** 10.1007/s10995-023-03698-1

**Published:** 2023-07-03

**Authors:** André Luís Bertani, Suzana Erico Tanni, Irma Godoy

**Affiliations:** Department of Internal Medicine, Botucatu Medical School, UNESP - Univ Estadual Paulista, Botucatu Campus, Pneumology Area - UNESP, Distrito de Rubião Junior S/N, Botucatu, São Paulo 18618-970 Brazil

**Keywords:** Pregnancy, Smoking, Tobacco, Waterpipe, E-cigarette

## Abstract

**Objectives:**

Our aim was to assess the use of dual/poly tobacco in a sample of pregnant women. Design: cross-sectional survey.

**Methods:**

Twenty prenatal care units in Botucatu, Sao Paulo, Brazil. We evaluated 127 high-risk pregnant smokers during prenatal care. Those who were 12–38 weeks pregnant and were currently smoking conventional cigarettes. The study enrollment took place between January 2015 and December 2015. The dual/poly prevalence of tobacco products during pregnancy and the characteristics related to smoking in pregnant smokers through a specific questionnaire containing questions related to sociodemographic characteristics, comorbidities, gestational history, smoking history, secondhand smoke exposure, nicotine dependence, motivation stage and use of alternative forms of tobacco.

**Results:**

Mean age was 26.9 ± 6.6 years, most had only elementary education and belonged to lower income economic groups. Twenty-five (19.7%) smoked conventional cigarettes only while 102 used conventional and alternative forms of tobacco products. Smoking pack-years was significantly lower in those only smoking conventional cigarettes than in dual/poly users. Proportion of patients with elevated degree of nicotine dependence was higher in conventional cigarettes users. On the other side, alcohol intake was higher in dual/poly smokers when compared to conventional cigarettes group. The alternative forms of smoking were associated with significantly higher occurrences of comorbidities as pulmonary, cardiovascular and cancer.

**Conclusions for Practice:**

The prevalence of alternative forms users of smoking products is high during pregnancy. These data reinforce the importance of a family approach towards smoking in pregnant women and education about the risks of alternative forms of tobacco.

## Significance

*What is Already Known?* Studies show that the prevalence of pregnant women who consume alternative forms of tobacco is high, and that these types of smoking are also harmful to pregnancy.

*What this Study Adds?* Our study describes the characteristics related to smoking in pregnant smokers and identifies dual/poly use of tobacco products.

## Introduction

The negative health consequences of smoking are well known; however, some effects are particularly important for women. Smoking is associated with premature aging of the female reproductive system leading to infertility and early menopause (Lumley et al., 2009; Reichert et al., 2008). Complications during pregnancy associated with both women and fetus, such as increased occurrence of placenta previa, ectopic pregnancy, miscarriage and sudden infant death syndrome make the harm even more significant (Ribot et al., 2014).

The prevalence of active smoking during pregnancy varies between countries. Data from USA, show that 7.2% of all women and 10.7% of those aged 20–24 who gave infants smoked cigarettes during pregnancy (Drake et al., 2018). In Brazil, a nationwide hospital-based study performed between 2011 and 2012 found that the prevalence of active smoking any time during pregnancy was 9.6% (Domingues et al., 2019). However, the prevalence of alternative forms of tobacco in this special group is still unknown, and in the case of waterpipe, is limited to Middle Eastern countries (Abdulrashid et al., 2018; Knishkowy & Amitai, 2005). Some studies in countries such as Jordan and Iran have shown prevalence rates of waterpipe to be around 5 to 8.0% in pregnant women and that it is an important risk factor for low infant weight (Akl et al., 2011; Al-Sheyab et al., 2016; Nematollahi et al., 2018). In Brazil, we did not identify any surveys evaluating the prevalence of alternative forms of tobacco smoking in pregnant women. Therefore, the aim of this study was to describe the characteristics related to smoking in pregnant smokers and identify dual/poly use of tobacco products.

## Methods

### Setting

This was a secondary analysis of a longitudinal study to test interventions to smoking cessation in a convenience sample of pregnant smokers followed at twenty prenatal care units in Botucatu, Sao Paulo, Brazil. The applied methodology was the same used in the previous study, where further details can be found (Bertani et al., 2021).

This is a cross-sectional study, and the study period was from January 2015 to December 2015.

### Participants and Questionnaire

Briefly, the main inclusion criteria were to be current smokers of conventional cigarettes during pregnancy (12–38 weeks pregnant). Smoking status was confirmed by measuring carbon monoxide (CO) in exhaled air by a standardized technique (Micro CO Meter, ^©^Cardinal Health, England, UK). One hundred and forty-three pregnant women were invited to participate and 127 signed the Informed Consent Form and were included in the study. Sixteen pregnant women refused to participate in the study. Questions including general characteristics, gestational and smoking history, secondhand smoking exposure, the Fagerström test for nicotine dependence (Heatherton et al., 1991), stage of motivation and concomitant use of alternative forms of tobacco were included in a questionnaire (Bertani et al., 2015). An alternative and common form of smoking in Brazil, mainly in rural areas, is a straw cigarette made with rustic tobacco hand-rolled in a straw sheet (Xavier et al., 2018). Therefore, we considered the straw cigarette as an alternative form of tobacco use as demonstrate in the results (Table 1). Patients were asked about smoking associated comorbidities and reported medical conditions were checked in their medical records. Socioeconomic status was obtained by applying a specific assessment scale for Brazil—Brazil Economic Classification Criteria (Pesquisa ABEP, 2012). According to this scale higher scores are associated with better socioeconomic status.

**Table 1:** Characteristics of pregnant women according the use of tobacco form

Variables	CC	CC Plus AF	p
	n = 25/19.7%	n = 102/80.3%	
Age (years)	28(18–33)	28(21–32)	0.328
Marital status N (%) Married	21(84.0)	64(62.7)	0.074
Initiation age (years)	14(13–15.5)*	13(11–14.2)	**0.005**
Smoke-load (pack-years)	5.4(3.2–14.5)	19.5(9.7–42.2)*	** < 0.001**
Cessation Attempts n (%)	17(68.0%)	58(56.8%)	0.431
Secondhand Smoke n (%)	20(80.0%)	73(71.5%)	0.548
Motivational Stage n (%)			
Pre-contemplative	9(36.0%)	43(42.2%)	0.738
Contemplative	16(64.0%)	59(57.8%)	
Nicotine Dependence n (%)			
Mild	3(12.0%)	35(34.3%)*	
Moderate	5(20.0%)*	7(6.9%)	**0.026**
High	17(68.0%)*	60(58.8%)	
Socio-economic classification*	19(15–22)*	15(12–21)	**0.024**
Comorbidities (%)			
Pulmonary	3(12.0%)	53(51.9%)*	
Cardiovascular	12(48.0%)	75(73.5%)*	**0.003**
Cancer	0(0.0%)	41(40.1%)*	
Other**	11(44.0%)*	38(37.2%)	
Alcohol intake (%)***	2(8.0%)	53(51.9%)*	** < 0.001**
Smoking in previous pregnancies (%)	17(68.0%)	81(79.4%)	0.341
Miscarriage (%)	8(7.8%)	51(50.0%)	0.163

Bold values indicate *p* < 0.05

*CC* Conventional Cigarettes, *AF* Alternative Forms

*Higher score represent better socio-economic condition

**Diabetes Mellitus, Dyslipidemia, Hypothyroidism and Lupus. Values expressed in median (1st and 3rd quartile) and proportions. p < 0.05 evaluated by Kruskal–Wallis One Way Analysis of Variance on Ranks, Dunn’s method, Chi-Square and Fisher’s exact test. Values expressed as proportions. a, b: different letters indicate a statistically significant difference between groups in each category (lines)

***Any intake (quantity and type of drink) concomitant with the gestational period

The author had access to medical records with a private password and the participants received numbers to preserve their data.

All methods were performed in accordance with the relevant guidelines and regulations. Informed consent was obtained from all participants.

The trial was registered at www.ensaioclinicos.gov.br (Registration number RBR-72j2yh).

### Statistical Analysis

Descriptive statistics were used to describe the features of all participants. Chi-Squared and Fisher’s Chi-squared tests were used to compare values of categorical variables and presented as frequency and percentage. Analysis of variance (ANOVA) followed by the Tukey or Kruskal–Wallis test followed by Dunn's test was used to compare demographic, socioeconomic condition and general characteristics and presented as medians and quartiles. Statistical analysis used SigmaPlot 12.0. and statistical significance was considered when p < 0.05.

This was a secondary analysis of a longitudinal study that tested a smoking cessation intervention. However, the sample size for this cross-sectional study was not performed as we used sample data from the main study.

## Results

Eligible participants were 127 and the results are presented below. Demographic characteristics of participants, according to dual use of alternative forms of tobacco, are presented in Table 1. A minority of the pregnant women (19.7%) smoked only conventional cigarettes and 80.3% used conventional cigarettes associated with at least one alternative forms of tobacco (waterpipe, straw cigarette, e-cigarette, and Bali cigarette). As each of the pregnant women could use one or more of these alternative forms of tobacco, we separated the pregnant women into only two groups to facilitate understanding (conventional cigarettes and dual/poly users). Twenty-three pregnant women used waterpipe, 69 straw cigarette, 30 e-cigarette and 28 used Bali cigarettes. Age of initiation was statistically lower, and the smoke-load was statistically higher for dual/poly users of tobacco when compared to conventional cigarettes. Mild degree of nicotine dependence was higher in the alternative forms group (34.3%); in contrast, moderate (20%) and high (68%) degree were higher in the conventional cigarette group.

According the socioeconomic classification scale score, women using conventional cigarettes presented better socioeconomic status when compared to those using alternative forms of smoking (19 (15–22) vs 15 (12–21), p = 0.024).

Report of comorbidities including pulmonary, cardiovascular and cancer were present in a higher proportion in users of dual/poly forms of smoke than in isolated conventional cigarettes group. This also true for alcohol intake during current pregnancy.

## Discussion

The main finding of this study was a high prevalence of the combined use of conventional and alternatives forms of smoking by pregnant women. The use of combined forms of smoking was associated com a lower age of smoking initiation, degree of nicotine dependence, socioeconomic classification, and higher smoke-load and alcohol intake during pregnancy. An important finding was a higher prevalence of reported comorbidities in pregnant women in users of alternatives forms of smoking. It also showed that smokers pregnant women are exposed to a high rate of secondhand smoking, a history of smoking in previous pregnancies and miscarriage as showed in previous studies (Da Motta et al., 2010).

One of the potential limitations of this study is that majority of the sample was taken from an outpatient clinic that treats high-risk pregnant women. It is also important to point out that this is not a prevalence study but a single center study. However, the sample characteristics show the importance of investigating all forms of tobacco product use during pregnancy everywhere regardless if the sale, importing and advertising of any devices for smoking is not or not permitted.

Our data show that more than 80% of pregnant smokers studied used alternative forms of tobacco. In the state of Sao Paulo, Brazil, an observational study evaluating the knowledge of pregnant women on tobacco use and alternative forms of smoking showed that 41% had some experience with flavored cigarettes and 19.7% with waterpipes (Bertani et al., 2015). In our study 18% of pregnant smokers reported dual use of waterpipes and conventional cigarettes. A Brazilian national survey showed that the consumption of straw cigarettes and waterpipe increased between the years 2013 and 2019. During pregnancy, a study performed in Jordan evaluating 285 women and anthropometric measurements of live infants showed that 26% used waterpipes, 29% conventional cigarettes only, and 9% used both during pregnancy (Al-Sheyab et al., 2016). Between 2016 and 2018, 714 women were assessed in southeastern Iran; 8.2% of them smoked waterpipes during gestation (Nematollahi et al., 2018). A systematic review of literature evaluating the prevalence of waterpipe tobacco use in the general and specific populations showed that 5–6% of pregnant women reported smoking waterpipes during pregnancy (Akl et al., 2011).

No international or national studies on the prevalence of straw cigarette use during pregnancy was found. However, a study evaluating the prevalence of tobacco product use in the south region of Brazil showed that 56.7% of women interviewed smoked conventional and straw cigarettes concomitantly (Scarinci et al., 2012).

The mean age of women in our study was under 30 years. Most lived in a stable union, had only elementary education, belonged to lower income classes, and had smoked during previous pregnancies. In agreement with our data, a study conducted in six Brazilian capitals showed that the mean age of pregnant smokers were under 30 years old, 87.8% lived in secondhand smoking conditions, 82.3% smoked in previous pregnancies, and 57.5% had only primary education (Kroeff et al., 2004). Previous study, identified that 51.4% had smoked during previous pregnancies (Da Motta et al., 2010). Although well-known in literature, the above information is still relevant in guiding smoking cessation strategies in women who smoke during pregnancy.

Studies in literature with pregnant smokers have evaluated tobacco-related diseases, demographic data and factors associated with smoking and smoking cessation during gestation and after delivery. However, no studies evaluating comorbidities in pregnant smokers was found. In our study, we easily obtained comorbidity data because a proportion of the pregnant women attending the outpatient clinic did so due to the presence of a pregnancy associated comorbidity.

In conclusion, the prevalence of pregnant women who smoke conventional cigarettes as well as other tobacco products is high. Smoking in previous pregnancies, alcohol consumption during the current pregnancy, lifetime tobacco exposure, cessation attempts, and degree of nicotine dependence may be linked to dual/poly use of tobacco products during pregnancy. Although, social characteristics are very important in the initiation and maintenance of the dependence, we can speculate that alternative forms of tobacco may have contributed for smoking during gestation. These data reinforce the importance of a family approach towards smoking in pregnant women and education on the risks of alternative forms of smoking.

## Data Availability

Not applicable.
